# Bruton’s Tyrosine Kinase Inhibitors (BTKIs): Review of Preclinical Studies and Evaluation of Clinical Trials

**DOI:** 10.3390/molecules28052400

**Published:** 2023-03-06

**Authors:** Dariusz Rozkiewicz, Justyna Magdalena Hermanowicz, Iwona Kwiatkowska, Anna Krupa, Dariusz Pawlak

**Affiliations:** 1 Department of Pharmacodynamics, Medical University of Bialystok, Mickiewicza 2c, 15-222 Bialystok, Poland; 2 Department of Clinical Pharmacy, Medical University of Bialystok, Mickiewicza 2c, 15-222 Bialystok, Poland; 3 Department of Internal Medicine and Metabolic, Medical University of Bialystok, M. Sklodowskiej-Curie 24a, 15-276 Bialystok, Poland

**Keywords:** Bruton’s tyrosine kinase inhibitors, Bruton’s tyrosine kinase, cancer, solid tumor, autoimmune disease

## Abstract

In the last few decades, there has been a growing interest in Bruton’s tyrosine kinase (BTK) and the compounds that target it. BTK is a downstream mediator of the B-cell receptor (BCR) signaling pathway and affects B-cell proliferation and differentiation. Evidence demonstrating the expression of BTK on the majority of hematological cells has led to the hypothesis that BTK inhibitors (BTKIs) such as ibrutinib can be an effective treatment for leukemias and lymphomas. However, a growing body of experimental and clinical data has demonstrated the significance of BTK, not just in B-cell malignancies, but also in solid tumors, such as breast, ovarian, colorectal, and prostate cancers. In addition, enhanced BTK activity is correlated with autoimmune disease. This gave rise to the hypothesis that BTK inhibitors can be beneficial in the therapy of rheumatoid arthritis (RA), systemic lupus erythematosus (SLE), multiple sclerosis (MS), Sjögren’s syndrome (SS), allergies, and asthma. In this review article, we summarize the most recent findings regarding this kinase as well as the most advanced BTK inhibitors that have been developed to date and their clinical applications mainly in cancer and chronic inflammatory disease patients.

## 1. Introduction

Protein kinases catalyze the phosphorylation of proteins, which changes their activity or ability to interact with other molecules, affecting, e.g., cellular growth, differentiation, survival, and proliferation. In addition, kinases are involved in several signal transduction cascades in which signals are carried from the cell membrane to the cytoplasm and nucleus. Therefore, protein kinase activity dysregulation plays a crucial role in the pathogenesis of a number of diseases, including autoimmune, cardiovascular, neurological, and inflammatory diseases, as well as a variety of cancers [1]. As a result, this enzyme family, which consists of 518 members, has risen to the status of one of the most significant drug targets in the twenty-first century [2]. One of these kinases is Bruton’s tyrosine kinase (BTK), which is receiving constantly growing attention since inhibitors of this kinase (BTKIs) have demonstrated remarkable anticancer effects in clinical investigations. When we take into consideration the fact that cancer is the second leading cause of death for people under the age of 70 in 112 of the 183 countries and the third or fourth leading cause of death in 23 of the other nations [3], it becomes abundantly clear that every effort that brings researchers closer to developing a more effective cancer treatment is urgently required. This article provides a concise summary of recent research on BTKIs and their therapeutic potential. In addition, it highlights some of the preclinical studies and clinical trials that have been conducted, as well as discusses current limitations and future aspirations.

## 2. Bruton’s Tyrosine Kinase

### 2.1. Molecular Structure of BTK

**Figure 1:**
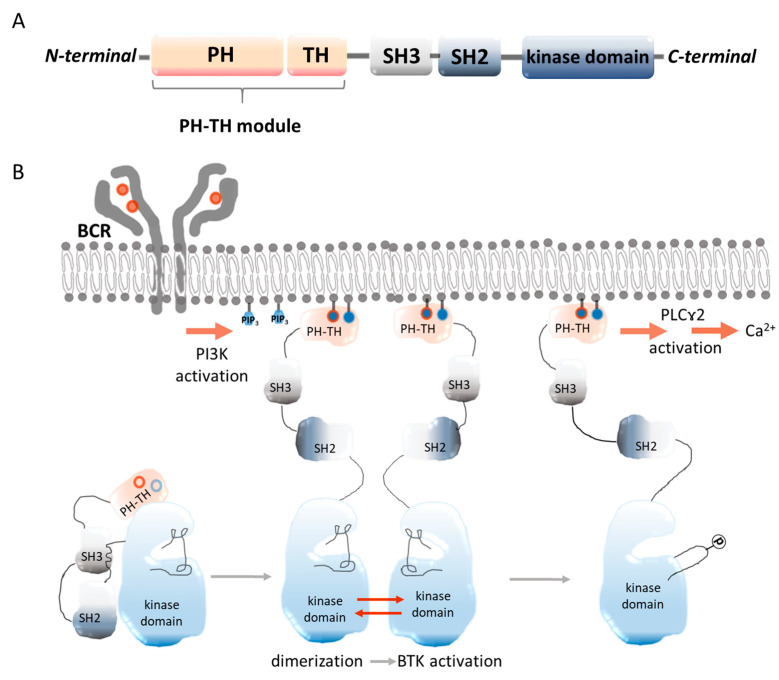
Schematic representation of the BTK structure. (**A**) PH**—**the pleckstrin homology domain, which has the capacity to bind phospholipids, allowing BTK to be recruited from the cytosol to the plasma membrane. TH—the Tec homology domain, which is required for the stability of BTK. SH3, SH2**—**Src domains are important in protein–-protein interactions. SH2 is a phosphoamino acid binding domain that specifically recognizes phosphotyrosine residues. Kinase domain—the protein’s catalytic domain [7]; BTK activation in the B-cell receptor (BCR) pathway. When the SH3 domain of BTK binds to the SH2-kinase linker, it locks the kinase domain into an inactive conformation, resulting in a compact and autoinhibited Src-like module of BTK. Both the assembled conformation of the Src-like module of BTK and the inactive conformation of the kinase domain are stabilized by the PH-TH module. In the next step, the PH-TH module binds to two PIP3 lipids, which triggers the dimerization of the BTK PH-TH module on the membrane in a switch-like manner. This in turn activates BTK by trans-autophosphorylation [8,9] (**B**).

Understanding the molecular structure of BTK is essential for deciphering BTK’s involvement in cell signaling and might aid in the development of BTK-specific inhibitors. BTK is a non-receptor tyrosine kinase (NRTK) and belongs to the Tec family of kinases (TFKs), which are distinguished by the inclusion of an N-terminal pleckstrin homology domain (PH), a proline-rich TEC-homology domain (TH), SRC-homology 3 (SH3), 2 (SH2) domains, and a C-terminal tyrosine kinase domain [4,5,6] as shown in Figure 1.

**Figure 2:**
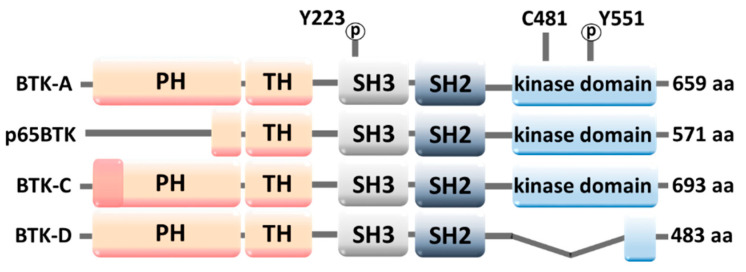
Structures of BTK isoforms. pY223 and pY551 are activating phosphorylation sites; C481 is a binding site for BTK inhibitors ibrutinib, spebrutinib, and acalabrutinib [10]. In this review, the term “BTK” refers to the BTK-A isoform unless it is clearly stated otherwise.

Recent studies have revealed that there are several isoforms of BTK (Figure 2): BTK-A, p65BTK, BTK-C, and BTK-D, which differ in molecular structure. p65BTK has a partial PH domain; BTK-C has an extended PH domain; and BTK-D has a partial kinase domain [6,10].

### 2.2. Occurrence of BTK and Its Role

**Figure 3:**
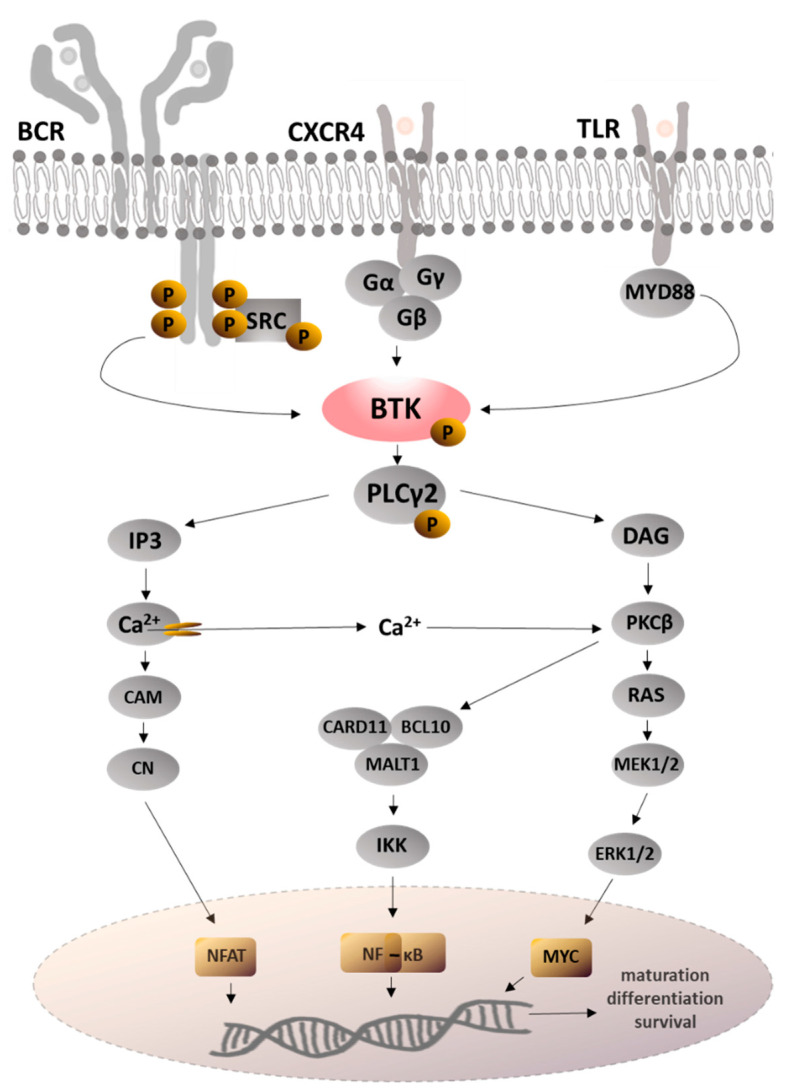
BTK pathway. BCL10—B-cell lymphoma/leukemia 10 protein, BCR—B cell receptor, CAM—calmodulin, CARD11—caspase recruitment domain-containing protein 11, CN—calcineurin, CXCR4—chemokine receptor type 4, DAG—diacylglycerol, ERK1/2—extracellular signal-regulated protein kinase ½, IKK—inhibitor of NF-κB kinase, IP3—inositol 1,4,5-trisphosphate, MALT1—mucosa-associated lymphoid tissue lymphoma translocation protein 1, MAPK—mitogen-activated protein kinase, MEK1/2—MAPK/ERK kinase ½, MYC—transcription factor, MYD88—myeloid differentiation primary response protein 88, NFAT—nuclear factor of activated T-cells, NF-кB—nuclear factor kappa-light-chain-enhancer of activated B-cells, PKCβ—protein kinase C β, PLCγ2—phospholipase Cγ2, RAS—rat sarcoma virus GTPase, SRC—protooncogene tyrosine-protein kinase, TLR—toll-like receptor.

All hematopoietic cells express BTK-A, but it is primarily found in B lymphocytes, where it plays an important role in maturation, differentiation, and survival, as well as cell signaling [11,12]. The B-cell receptor (Figure 3) activates phosphoinositide 3-kinase (PI3K), which catalyzes the production of phosphatidylinositol-3,4,5-triphosphate (PIP3) [5,9]. The pleckstrin homology domain of BTK binds PIP3 to phosphatidylinositol in a cell membrane, therefore recruiting BTK to the membrane through the PH-TH module [13]. Binding BTK promotes PIP3 to phosphorylate phospholipase Cγ2, which in turn hydrolyzes phosphatidylinositol 4,5-bisphosphate (PIP2) to the two second messengers: inositol 1,4,5-trisphosphate (IP3) and diacylglycerol (DAG) [14,15]. IP3 activates calcium channels, allowing nuclear factor of activated T cells (NFAT) to enter the nucleus, and DAG activates protein kinase C β (PKCβ), which stimulates nuclear factor kappa-light-chain-enhancer of activated B-cells (NF-kB) pathway factors. NFAT and NF-kB are important for B-cell survival, proliferation, chemokine, and cytokine production [7] (Figure 3). In addition, Tec family tyrosine kinases can activate C-X-C chemokine receptor type 4 (CXCR4), which affects tumor growth, survival, and migration [16]. In turn, inhibiting BTK suppresses BCR signaling and causes apoptosis by decreasing antiapoptotic B-cell lymphoma 2 (BCL-2), B-cell lymphoma extra large (BCL-XL), and myeloid cell leukemia 1 (Mcl-1) protein [17,18]. Aside from its initial role in BCR signaling, it has been demonstrated that BTK is involved in transmitting signals from a wide range of receptors, e.g., Toll-like receptors (TLRs) in B-cells, Fc-gamma receptor (FCγR) or toll-like receptors (TLRs) in macrophages or plasmacytoid dendritic cells (pDCs), mast cells, and basophils [5,11,19]. In addition, BTK is essential for the synthesis of proinflammatory cytokines such as tumor necrosis factor α (TNFα) and interleukin-1β (IL-1β), as well as for the process of degranulation and the release of histamine [20]. Recent research has shown that BTK is responsible for regulating the nucleotide-binding oligomerization domain-like receptor protein 3 (NLRP3) inflammasome [21,22] by modifying subcellular localization and inflammasome assembly [23]. The NLRP3 inflammasome is a cytosolic multiprotein signaling complex that can activate proinflammatory cytokines and chemokines such as interleukin-1 (IL-1), interleukin-18 (IL-18), and gasdermin D (GSDMD), thereby directing the inflammatory response. It also acts as a platform for caspase-1 activation, leading to cell death [24,25,26]. Consistently, BTK inhibitors reduce IL-1 production and prevent NLRP3 inflammasome activation in septic animals [27]. The inflammasome is involved in a number of acute and chronic conditions, including myocardial infarction, stroke, inflammation of the liver, type 2 diabetes, Alzheimer’s disease, Parkinson’s disease, and sepsis [24,28,29,30]. Thus, BTKIs are currently becoming even more attractive as potential therapeutic targets. 

Other BTKs appear to be mostly expressed in tissues and malignancies that are not associated with B-cells. p65BTK is overexpressed in colon carcinoma cell lines and tumors [31,32,33], lung, and ovarian cancers [32,34]. Additionally, the levels of p65BTK expression in patients with ovarian cancer are associated with early recurrence as well as shorter progression-free survival, both of which are signs of resistance to therapy [32]. Similarly, Lavitrano et al. found that p65BTK is present in the great majority of colon carcinomas, where its expression correlates with histotype and cancer development [33,35]. Additionally, p65BTK expression was substantially higher in epidermal growth factor receptor (EGFR) wild-type adenocarcinomas [34]. On the other hand, BTK-C has been found in breast and prostate cancer cells, where it impacts apoptosis resistance, therapeutic escape, and glucose uptake [10,33,36,37]. Recently, unusually high expression of p65BTK and BTK-C has been seen in patient specimens of oral squamous cell carcinoma (OSCC), and treatment with ibrutinib has been found to decrease migration and invasion in these malignancies [38,39]. Neuroblastoma cells also express BTK, and high expression corresponds with poor neuroblastoma relapse-free survival [33,40,41]. These findings suggest that different types of BTK can be therapeutic targets in solid tumors. Due to the recent discovery of p65BTK and BTK-C, further study is required to determine their roles. 

### 2.3. BTK Down- and Upregulation

BTK downregulation is responsible for X-linked agammaglobulinemia (XLA), a severe primary immunodeficiency condition caused by mutations in the BTK gene, which inhibits precursor B-cells in the bone marrow from maturing, resulting in B lymphocyte insufficiency and infection vulnerability [42,43]. It also decreases 12-O-tetradecanoylphorbol-13-acetate (TPA)-induced matrix metalloproteinase-9 (MMP-9) expression and MCF-7 breast cancer cells invasion and metastasis [44]. By using triplet BTK short interfering RNA duplexes (siRNA), Heinonen et al. were able to downregulate BTK expression in the RBL-2H3 mast cell line and hence inhibit histamine release [45]. This suggests that BTK kinase activity is essential for proper mast cell activation and may play a role in determining allergic reactions. On the other hand, multiple B-cell cancers, such as chronic lymphocytic leukemia (CLL), mantle cell lymphoma (MCL), diffuse large B-cell lymphoma (DLBCL), and acute myeloid leukemia (AML), are linked with the upregulation of BTK [13,46]. The hematological system has been the primary focus of the majority of studies that have been reported on BTK; however, the role of BTK in solid tumors is yet relatively unknown. Nonetheless, a growing amount of experimental and clinical evidence has shown in recent years the importance of BTK not only in B-cell malignancies, but also in solid tumors, such as breast, ovarian, colorectal, prostate [47,48,49,50,51,52], esophageal, gastric and bladder cancer [6,49,53,54]. Notably, in prostate cancer cells, reducing the expression of BTK-C by RNAi or inhibiting its activity using BTK-specific inhibitors such as ibrutinib, AVL-292, or CGI-1746 leads to a decrease in cell survival. Inhibiting BTK under these conditions results in an increase in the expression of genes related to apoptosis, while overexpression of BTK-C is associated with an increase in the expression of genes whose functions are related to cytoskeletal structure, cell adhesion, and the extracellular matrix [51]. These findings are of particular interest given a recent report by Z. Zhu et al. that showed that invasive prostate cancer with bone metastasis exhibited higher BTK expression than non-invasive prostate cancer or benign prostatic hyperplasia. In in vitro tests using prostate cancer cell lines, the BTK inhibitor ibrutinib was found to drastically reduce prostate cancer cell proliferation, wound healing, migration, and invasion, as well as block tumor cell matrix metalloproteinase-2 (MMP-2) and MMP-9 protein production [55]. Zucha et al. also reported that cancer stem-like cells (CSCs) in ovarian carcinoma overexpress BTK, and this mechanism is responsible for resistance to cisplatin. BTK silencing significantly decreased the expression of the Janus kinase 2 (JAK2)/signal transducer and activator of transcription 3 (STAT3) and, in consequence, reduced cancer cell viability via the SRY-box transcription factor 2 (Sox-2) and BCL-XL genes and increased susceptibility to cisplatin [56]. In addition, it has been documented that BTK is overexpressed in gastric carcinoma cells but not in normal gastric mucosa epithelial cells. Therefore, BTK expression knockdown preferentially affects the development of gastric cancer cells, but not normal cells [54]. Giordano et al. demonstrated that p65BTK expression is controlled by the activation of the RAS/MAPK pathway by finding that both its expression and activity are downregulated in non-small cell lung cancer (NSCLC) cells treated with the MEK inhibitor trametinib [34].

## 3. Bruton’s Tyrosine Kinase Inhibitors

Due to BTK’s significance in B-cell survival and proliferation, as well as its overexpression in various B-cell malignancies, it has been identified as a possible therapeutic target for the treatment of leukemias and lymphomas. Since the discovery and development of the BTKIs, the possibility of conventional chemotherapy-free management of B-cell malignancies has been introduced, which has revolutionized therapeutic strategies. 

Beyond this, BTK is expressed in macrophages, plasmacytoid dendritic cells, mast cells, basophils, neutrophils [12], and other types of immune cells; hence, inhibiting BTK signaling may have an effect on innate and adaptive immunity. In particular, mast cell Fc-epsilon receptor (FcεR) signaling is likewise regulated by BTK, making BTK inhibitors a potential treatment for immunoglobulin E (IgE)-related disorders such as allergies, asthma, and dermatitis [11]. Additionally, Corneth et al. demonstrated that enhanced BTK activity is correlated with autoimmune disease, which is linked to autoantibody and inflammatory mediator formation. This finding shows that B-cell–targeted treatments have potential in the therapy of RA, systemic lupus erythematosus (SLE), MS, and Sjögren’s syndrome (SS) [7,57,58,59]. The genetic correlations of RA, MS, and SLE biomarkers with members of the TEC family were analyzed, and the results indicated that BTK and members of the TEC family may not be disease drivers, but they are part of signaling pathways engaged in the pathophysiology of autoimmune disorders [12]. Given that BTKIs decrease the levels of proinflammatory cytokines, it has been hypothesized that BTKIs may minimize the excessive and damaging immune response in severe COVID-19 infection and the consequent respiratory problems [60,61,62,63]. It was revealed in research that was carried out on 2902 patients coming from six different treatment centers for CLL patients that the symptoms of COVID-19 had emerged in less than one percent of patients who were receiving ibrutinib. It has also been found that it had a protective effect against the development of severe types of COVID-19 in patients who had CLL [14]. 

BTKIs can be either irreversible (presence of the Michael acceptor moiety that can form a covalent bond with the conserved Cys481 residue) or reversible (presence of a weak, reversible hydrogen bond or a hydrophobic interaction) [5,20]. To date, five drugs capable of irreversibly inhibiting BTK have been commercialized, whereas other reversible BTKIs are undergoing preclinical and clinical investigations mainly for the long-term treatment of autoimmune diseases, especially RA and MS. Recently, there have been developed hybrid BTKIs with the ability to establish reversible covalent bonds with the Cys481 residue and temporarily inactivate the enzyme, e.g., PRN1008 (rilzabrutinib). Combining the benefits of covalent and non-covalent inhibitors, this class of inhibitors is highly effective and selective, with fewer off-target effects [20,64]. Reversible covalent inhibitors are well suited for therapeutic applications that call for prolonged target engagement and/or quicker target disengagement due to their tunability of dissociation rates and inhibitor residence duration. High and sustained BTK occupancy was maintained in rats even when rilzabrutinib plasma concentrations approached the lower limit of detection, confirming rilzabrutinib’s considerable cellular occupancy in vivo [65]. This results in very low systemic exposure, which increases tolerance and decreases the likelihood of side effects while maintaining efficacy over long periods of time. New research shows that rilzabrutinib inhibits the development of autoantibodies through a number of different mechanisms, including immediate anti-inflammatory effects, neutralization of pathogenic autoantibody signaling, and suppression of new autoantibody formation in innate and adaptive immune cells [65,66].

### 3.1. Approved Irreversible BTKIs

**Figure 4:**
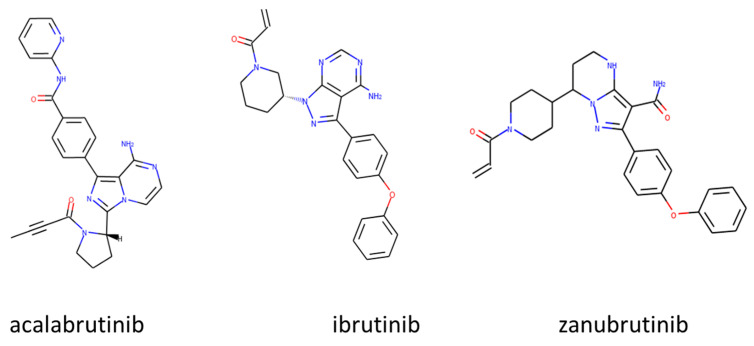
BTKIs approved by the FDA [67,68,69].

Currently, the U.S. Food and Drug Administration (FDA) has approved three BTK inhibitors: ibrutinib (2013), acalabrutinib (2017), and zanubrutinib (2020) (Figure 4). Tirabrutinib (2020) and orelabrutinib (2020) are two other BTK inhibitors that have been approved by the Japan Pharmaceuticals and Medical Devices Agency and the China Food and Drug Administration, respectively. 

#### 3.1.1. Ibrutinib (Imbruvica^®^) 

Ibrutinib is an orally administered, effective, irreversible first-generation BTK inhibitor that covalently binds to a cysteine residue (Cys-481) near the ATP-binding pocket of BTK [70] (Table 1). As a result, it inhibits BCR signaling and downregulates NF-кB signaling, drastically decreasing tumor growth and boosting apoptosis in the process [71]. Besides BTK inhibition, it also affects other TEC family kinases, such as interleukin-2-inducible T-cell kinase (ITK), and therefore modifies cell adhesion within the tumor microenvironment as well as modulates chemotaxis and cell-to-cell signaling [42]. ITK expression is mostly restricted to T-cells and is essential for the growth and proliferation of T lymphocytes, activating downstream effectors in the TCR signaling pathway, similar to BTK in the BCR signaling pathway [72]. Since ITK promotes Th2 differentiation, blocking it may increase Th1 differentiation and, thus, the antitumor response, which has been proposed as a useful strategy in cancer therapy [73,74]. 

Ibrutinib was the first BTKi authorized by the FDA in 2013 [15]. In 2021, ibrutinib overtook the sales of all other cancer drugs to become the world’s fourth best-selling medication [79]. Ibrutinib is used to treat CLL, small lymphocytic lymphoma (SLL), Waldenström’s macroglobulinemia (WM), marginal zone lymphoma, and graft-versus-host disease [75,76,80,81,82,83,84,85]. In August 2022, ibrutinib was authorized in the United States for use in adult and pediatric patients aged 1 year and older with chronic graft-versus-host disease (cGVHD) after the failure of one or more systemic therapies; it is the first drug licensed for use in this age range [86]. Apart from BTK inhibition, ibrutinib also blocks the activation of EGFR, human epidermal growth factor receptor 2 (HER2), Erb-B2 receptor tyrosine kinase 3 (ErbB3), and Erb-B2 receptor tyrosine kinase 4 (ErbB4), which results in increased apoptosis of breast cancer cells. This indicates that ibrutinib has the potential to be a successful therapy, particularly for HER2+ breast cancer [37,87]. Another study has recently confirmed that ibrutinib is able to inhibit the progression and metastasis of breast cancer by stimulating the maturation of myeloid-derived suppressor cells (MDSCs) into dendritic cells (DCs), which induces antitumor immunity mediated by T-helper Type-1 (Th1) lymphocytes [88,89]. This is consistent with other research that found that BTK is expressed in MDSC, and ibrutinib leads to a considerable decrease in MDSC in mouse models of breast cancer and melanoma [90]. Notably, Gunderson et al. found that in pancreas ductal adenocarcinoma-bearing mice, treatment with ibrutinib reprogrammed macrophages toward a Th1 phenotype, which stimulated cluster of differentiation 8-positive (CD8+) T-cell cytotoxicity and decreased tumor development, demonstrating that BTK signaling drives tumor immunosuppression. The authors concluded that this is dependent on crosstalk between B-cells and Fc-gamma receptor-positive (FcRγ+) tumor-associated macrophages. This crosstalk results in the programming of TH2-type macrophages via BTK activation in a phosphatidylinositol 3-kinase (PI3K)γ-dependent way [91]. 

Moreover, the therapy with ibrutinib also resulted in a substantial decrease in the expression of vascular endothelial growth factor (Vegf), MMP-9, and C-X-C motif chemokine ligand 1 (Cxcl1), all of which are known to play a key role in the processes of carcinogenesis, angiogenesis, and metastasis [89]. Another study has revealed that ovarian carcinoma cell proliferation and survival were significantly reduced by BTK inhibitors when tested both in vitro (using cell lines) and ex vivo (using cells newly dissociated from human xenografts and cancer cells originating from patients) [32]. Likewise, BTK inhibitors promote apoptosis in gastric carcinoma cells and reduce the growth of gastric tumor xenografts [54]. Ibrutinib has also been shown to promote autophagic cell death in glioblastoma by a mechanism that appears to be mediated by the inhibition of the serine/threonine-specific protein kinase/mammalian target of rapamycin (Akt/mTOR) signaling pathway [92]. 

In addition to BTK, ibrutinib inhibits other intracellular kinases, including B lymphoid tyrosine kinase (BLK), bone marrow kinase on chromosome X (BMX), non-receptor tyrosine kinase (TEC), ITK, and Janus kinase 3 (JAK3) [15]. A lack of selectivity causes several off-target side effects, i.e., skin and dermatological problems, allergic reactions, fever, lymphadenopathy, edema, albuminuria, diarrhea, bleeding, infection, headaches, and atrial fibrillation [20,76,93,94,95]. On the other hand, ibrutinib’s off-target inhibition of JAK3, ITK, and EGFR means that it can target oncogenic pathways other than BTK in tumor cells and act as a T-cell modulator in combination immunotherapy [96,97,98,99]. Ironically, the novel, highly selective BTKIs may not have these potentially valuable side effects of ibrutinib.

Apart from this, drug resistance emerges in 60% of patients treated with ibrutinib [76] as the cysteine at position 481 of the BTK protein mutates into serine [52,100,101,102,103,104,105,106,107]. Among others, these aspects drove the quest for combination therapy and new generations of BTKIs, which are more selective and therefore have fewer off-target side effects and reduced toxicity [101,108,109,110,111,112]. 

#### 3.1.2. Acalabrutinib (Calquence^®^)

Acalabrutinib is a second-generation BTK inhibitor approved by the FDA in 2017 and is indicated in relapsed/refractory MCL and CLL [76,113,114] (Table 1). Unlike ibrutinib, only BTK, BMX, and ErbB4 are inhibited at clinically significant doses. Therefore, acalabrutinib possesses a high degree of selectivity and could minimize the incidence of the targeted side effects [75]. Compared to ibrutinib, acalabrutinib has more favorable pharmacologic properties, such as a fast oral absorption rate, a shorter half-life, and fewer side effects [115]. Acalabrutinib has been used widely in clinical studies to treat B-cell malignancies, myelofibrosis, ovarian cancer, multiple myeloma, and Hodgkin lymphoma [75]. In a phase III trial, acalabrutinib and ibrutinib were compared for efficacy and safety in patients with previously treated CLL. The results showed similar progression-free survival (PFS) in both treatment groups and lower treatment discontinuation due to adverse events in patients receiving acalabrutinib compared to ibrutinib [116]. 

#### 3.1.3. Zanubrutinib (Brukinsa^®^)

Patients with MCL, WM, and CLL/SLL can now be treated with zanubrutinib, a highly selective [117,118], irreversible second-generation BTK inhibitor [76] which was approved by the FDA in 2019 [119] (Table 1). There is a preference for BTK over TEC, and the compound does not interfere with ITK activity [5]. Therefore, there are far fewer side effects with zanubrutinib than there are with ibrutinib, including atrial fibrillation, hypertension, and hemorrhage [75,120,121]. In a recent two-part, single-arm, multicenter phase I study, zanubrutinib was well tolerated, and its antitumor activity was clinically significant when administered as monotherapy at 160 mg twice a day or 320 mg once a day [120]. As a result, zanubrutinib has a better safety profile in patients with B-cell malignancies than ibrutinib. Moreover, progression-free survival was considerably longer with zanubrutinib than with ibrutinib in patients with relapsed or refractory CLL or SLL, and zanubrutinib was also linked with fewer cardiac adverse events [122]. A study has shown that a second-generation BTK inhibitor could alleviate the toxicities associated with traditional BTK inhibitors by minimizing off-target effects such as those on HER2 and TEC kinases. It was a breakthrough that could be a potential game-changer in the treatment of cancer. Furthermore, models utilizing data from the ASPEN trial comparing the costs of treating WM with zanubrutinib or ibrutinib in the United States revealed that zanubrutinib is a more cost-effective therapeutic choice than ibrutinib [123]. 

#### 3.1.4. Tirabrutinib (Velexbru^®^)

In 2020, tirabrutinib, was approved for the treatment of recurrent or refractory primary central nervous system lymphoma (PCNSL) [124], and later for WM and lymphoplasmacytic lymphoma by the Japan Pharmaceuticals and Medical Devices Agency. Tirabrutinib is more selective than ibrutinib [75,76] (Table 1). Tirabrutinib monotherapy at a daily dosage of 480 mg under fasting conditions showed a good effectiveness and tolerable safety profile in a phase II study (ONO-4059-05 study) in patients with treatment-naive and relapsed/refractory WM. Both the major response rate (MRR) and the progression-free survival (PFS) rate at 24 months were 92.6%, which are rates that are comparable to or even higher than those seen in previous BTK inhibitor trials, e.g., ibrutinib (MRR 78%, 18-month PFS 84%), zanubrutinib (MRR 77%, 18-month PFS 85%) [125,126].

#### 3.1.5. Orelabrutinib

Orelabrutinib was approved by China’s Food and Drug Administration in 2020 for the treatment of adult patients with MCL who had received at least one prior treatment, as well as adult patients with CLL/SLL [75,127] (Table 1). It is a new, irreversible, covalent BTK inhibitor that targets only BTK (>90% inhibition). Although the phase I/II trial showed that orelabrutinib was effective and safe in patients with R/R CLL/SLL, more research is needed to compare it to ibrutinib in this patient population and to assess its efficacy and safety in patients who are treatment naive [128]. Currently, it is being studied in clinical trials for lymphoid malignancies and autoimmune diseases [129].

CLL—chronic lymphocytic leukemia; CNS lymphoma—primary central nervous system (CNS) lymphoma; GVHD—graft-versus-host disease; MCL—mantle cell lymphoma; MZL—marginal zone lymphoma; NHL —non-Hodgkin lymphoma; SLL—small lymphocytic lymphoma; WM—Waldenström’s macroglobulinemia. IC_50_—half-maximal inhibitory concentration, the inhibitor concentration that causes a 50% decrease in enzyme activity. Various kinase activity tests are utilized to assess the selectivity of the inhibitors; hence, reported IC_50_ statistics are widely diverse; e.g., the IC_50_ values for ibrutinib against ITC range from 0.5 nM to 218 nM [5,76]. BTK—Bruton’s tyrosine kinase; ITK—interleukin-2-inducible T-cell kinase, TEC—cytoplasmic tyrosine kinase.

### 3.2. BTK Inhibitors in Clinical Trials

The site mutations of BTK affecting Cys481 and the gatekeeper residue Thr474 have limited the use of irreversible BTKIs. In this regard, reversible inhibitors that do not interact with Cys481 have been developed. Currently, all of them are under clinical or preclinical investigation. 

#### 3.2.1. Spebrutinib (CC-292)

Spebrutinib is an orally bioavailable, highly selective, and effective covalent BTK inhibitor [130,131] (Table 2). Patients with R/R CLL, B-NHL, or WM were included in phase I, multicenter, open-label, dose-escalation research, and the results showed that CC-292 is well tolerated as a daily oral monotherapy at dosages up to 1000 mg once daily or 500 mg twice daily. More than 90% BTK receptor occupancy was seen at both the 4 and 24 h time periods in patients taking CC-292 twice daily. However, compared to ibrutinib and acalabrutinib, its clinical activity, especially response duration, was lower [132]. In a separate phase II multicenter clinical investigation, the effectiveness and safety of spebrutinib were also assessed in patients with active RA. Spebrutinib inhibited B-cell proliferation more effectively than T-cell proliferation in vitro, and it also suppressed the generation and degranulation of lymphoid and myeloid cytokines, as well as osteoclastogenesis. Increases in total CD19+ and mature naive CD27^−^CD38^−^IgD^+^ B-cells and decreases in transitional CD27^−^CD38^+^ B-cells were found in individuals treated with spebrutinib. The median BTK occupancy in peripheral blood was 83%. Serum levels of chemokine ligand 13 (CXCL13), macrophage inflammatory protein-1β (MIP-1β), and the biomarker for bone resorption, carboxy-terminal collagen cross-linking telopeptide (CTX-I), were all significantly decreased after treatment with spebrutinib [133].

#### 3.2.2. Evobrutinib (M2951, MSC-2364447C)

The novel, highly selective, central nervous system-penetrating, irreversible BTK inhibitor evobrutinib strongly suppresses BCR- and Fc receptor-mediated signaling, which makes it a promising agent in the treatment of RA, MS, and other autoimmune diseases [19,134,135] (Table 2). By forming a covalent bond with BTK, evobrutinib is able to block its target for a considerable time after the drug has been cleared from the body [136,137]. Additionally, evobrutinib was very selective in an assay with 267 different kinases. At 1 μM, only two other kinases besides BTK were blocked by more than 80%, whereas ibrutinib blocked 25 off-targets by more than 80% at 1 μM [135]. Evobrutinib’s excellent selectivity for BTK over EGFR and other Tec family kinases implies that it may have a minimal risk of off-target side effects [19]. Phase II trials confirmed that evobrutinib was well tolerated in MS, RA, and SLE patients [134,138]. 

#### 3.2.3. Vecabrutinib (SNS-062)

Vecabrutinib is a non-covalent BTKi that is selective and reversible. Efficacy in preclinical investigations against BTK wild-type and the C481 mutant has been demonstrated [139,140] (Table 2). Recently, a phase Ib dosage escalation trial showed that vecabrutinib was well tolerated up to 410 mg twice daily, the highest dose investigated; nevertheless, the activity shown in BTKi-resistant patients at the dose levels studied was deemed inadequate for phase II expansion of this patient group [141]. However, preclinical investigation showed that combining vecabrutinib and venetoclax greatly increased therapeutic effectiveness, and significantly enhanced survival [142].

#### 3.2.4. Pirtobrutinib (LOXO-305)

Pirtobrutinib (LOXO-305) is a reversible, extremely selective inhibitor that binds non-covalently to wild-type BTK or kinase domain-mutant BTK [139,143,144] (Table 2). With more than 300-fold selectivity for BTK over 98% of other kinases, the risk of off-target toxicities is reduced. Additionally, it maintains greater than 90% BTK inhibition at trough, which ensures efficient target inhibition throughout the dosing interval, even in proliferative tumors [139]. Furthermore, with a peak level about 90 times higher and a retention in human plasma 2.5 times greater than that of ibrutinib, pirtobrutinib has a more desirable pharmacokinetic profile [144]. In the multicenter, open-label, phase 1/2 study, favorable safety and encouraging effectiveness were demonstrated in a variety of B-cell tumors, including previously treated CLL, MCL, WM, and follicular lymphoma, including patients with resistance or intolerance to covalent BTKIs. The maximum tolerable dose was not achieved, and there were no signs of dose-limiting toxicity [145,146]. It is expected that pirtobrutinib, when used as first-line BTK inhibitor treatment in relapsed MCL, will be more effective than the covalent BTK inhibitors (ibrutinib, acalabrutinib, or zanubrutinib) [147,148]. Therefore, there is currently ongoing randomized, open-label, worldwide phase III research comparing pirtobrutinib monotherapy to ibrutinib, acalabrutinib, or zanubrutinib in patients with previously treated, BTK inhibitor-naive MCL (NCT04662255). The purpose of the trial is to confirm the activity and safety of pirtobrutinib in patients with relapsed MCL and to evaluate differences in efficacy, safety, and tolerability [149,150].

#### 3.2.5. Fenebrutinib (GDC-0853)

Fenebrutinib is a selective, reversible BTKi (Table 2). It forms hydrogen bonds with K430, M477, and D539 instead of covalent bonds with the C481 residue and therefore is useful in individuals with C481 mutation [139]. There were just three off-target kinases inhibited out of the 286 tested. The measured IC50 values showed that it had BTK selectivity greater than 100-fold against BMX (153-fold), FGR (168-fold), and SRC (131-fold) [151]. Fenebrutinib has been effective in phase II investigations of individuals with RA, SLE, chronic spontaneous urticaria (CSU), and in the treatment of B-cell malignancies [152,153,154,155]. Furthermore, currently ongoing trials will provide valuable insight into the effectiveness of fenebrutinib in MS (NCT05119569, NCT04586023, and NCT04544449).

### 3.3. Combination Therapy

Concurrent inhibition of BTK and other survival-related kinases such as PI3Kδ, SYK, and mTOR has been shown to have synergistic effects and helped overcome the drug resistance problem, reduce the therapeutic doses and therefore side effects [106,156,157]. For instance, the combination of enzastaurin (a protein kinase C beta inhibitor) and ibrutinib (BTKI) synergistically triggered G1 phase arrest and increased apoptosis, and inhibited cell migration and invasion in comparison to monotherapy [158]. Another study has reported that acquired idelalisib (a PI3Kδ inhibitor) resistance is overcome by the combination of idelalisib and ONO/GS-4059 (BTKI) in diffuse large B-cell lymphoma [159]. Similarly, Li et al. demonstrated that cotreatment with everolimus (an mTOR inhibitor) and PLS-123 (BTKI) synergistically inhibits migration and invasion in MCL, and inhibits tumor growth in the Granta519 xenograft model by 84.8% [160]. Moreover, ibrutinib inhibits the development of neuroblastoma xenografts in nude mice, and the combination of ibrutinib with the anaplastic lymphoma kinase (ALK) inhibitor crizotinib increases the suppression [40]. BTK inhibitors are also able to sensitize drug-resistant tumor suppressor p53 protein (TP53)-null colon cancer cell lines, patient-derived organoids, and xenografts to 5-Fluorouracil (5-FU), as demonstrated by Lavitrano et al. [33]. When combined with cisplatin, ibrutinib showed a synergistic impact on chemotherapy and may be useful as an adjuvant to overcome cisplatin resistance in ovarian cancer [56]. Additionally, it was also shown by Giordano et al. that ibrutinib, AVL-292, and RN486 re-sensitize drug-resistant NSCLC to standard-of-care (SOC) chemotherapy (Cisplatin, Gemcitabine, and Pemetrexed), resulting in decreased cancer cell viability and severely inhibited cell proliferation and clonogenicity and thus giving hope for cancer patients with chemotherapy-resistance [34,161]. Recently, it has been found that ibrutinib-mediated combination immunotherapy with a sialic acid derivative-modified nanocomplex (SA-GA-OCT@PC) is more effective against solid tumors. Novel insights for tumor immunotherapy were gained from in vitro and in vivo research showing that SA-GA-OCT@PC successfully accumulated in tumor-infiltrating T-cells driven by Siglec-E and promoted Th1-dominant antitumor immune responses [162].

### 3.4. Dual Inhibitors

An alternative to combined therapy are dual inhibitors, which simultaneously block two different types of proteins, for example, BTK/PI3Kδ, BTK/JAK3, BTK/BMX, BTK/MAPK-interacting kinases (MNKs), BTK/FMS-like tyrosine kinase 3 (FLT3), and BTK/hematopoietic cell kinase (HCK) [6]. Most of them are under preclinical and clinical investigation. The characteristics of these substances are beyond the scope of this study and are left for future research.

## 4. Conclusions

BTK inhibitors have transformed the therapeutic landscape for leukemias and lymphomas. A rising amount of experimental and clinical evidence has shown that BTK is not only important in B-cell malignancies but also in solid tumors, autoimmune, and inflammatory diseases. Although the direct effects on BCR signaling pathways are well recognized, the pleiotropic implications of BTK inhibitors on the overall tumor microenvironment are only just beginning to be comprehended. It is mainly due to the fact that BTK is expressed not only in B-cells but also in other types of immune cells such as MDSC, dendritic cells, mast cells, and macrophages, all of which are components of the tumor microenvironment in solid tumors [15,163]. This cell-to-tumor cross-talk via signaling pathways is critical for tumor progression. Furthermore, it is crucial to understand the biological activities of each isoform of BTK and identify the key signaling pathways in which they are engaged [28]. Moreover, there are around 500 identified protein kinases in humans, all of which need ATP as their cofactor, making the inhibitors unspecific, and cross-reactivity might lead to undesired side effects as a result [5]. Finally, malignant cells’ genetic instability and gatekeeper mutations in the target protein kinase cause resistance to protein kinase treatments, which has led to the development of next-generation inhibitors. Although inflammatory processes are not inherently genetically unstable, it is unknown if acquired resistance develops throughout treatment [2]. There are hundreds of BTKIs being investigated, and a few whose safety and efficacy have been demonstrated in a range of malignancies, but the long-term efficacy of these treatments has yet to be determined because of the study’s short follow-up period. On top of that, several novel BTK inhibitors, including reversible drugs, do not yet have long-term clinical studies available.

In the future, we will also need to further investigate the combination therapy of BTK inhibitors with other anti-tumor drugs in order to increase the anti-tumor efficacy and prevent the adverse reactions that are caused by non-selective inhibition by BTK inhibitors. Additionally, given that acquired resistance-causing mutations have been shown to be present months before disease progression, screening for these variants may enable prompt therapy adaptation [164]. The appraisal of recent successes in the development of BTK inhibitors, despite the lack of entirely satisfying results, implies that continuous development of novel cancer-fighting strategies will bring us to a new generation of drugs and a better outcome for patients.

## Figures and Tables

**Table 1:** Summary of approved BTKIs [5,14,20,75,76,77,78].

Name	Synonyms	Type of Inhibitor	Indications	Year of Approval	Activity (IC_50_)
Ibrutinib Imbruvica^®^	CRA-032765 PC-32765 PCI-32765 PCI-32765-00	Irreversible	MCL, CLL, SLL, WM, MZL, GVHD	2013	BTK IC_50_ = 0.47 nM
					ITK IC_50_ = 55 nM
					TEC IC_50_ = 3.2 nM
Acalabrutinib Calquence^®^	ACP-196	Irreversible	CLL, SLL, MCL	2017	BTK IC_50_ = 2.5 nM
					ITK IC_50_ > 20,000 nM
					TEC IC_50_ = 37 nM
Zanubrutinib Brukinsa^®^	BGB-3111	Irreversible	NHL, CLL, MCL	2019	BTK IC_50_ = 0.3 nM
					ITK IC_50_ = 56 nM
					TEC IC_50_ = 2 nM
Tirabrutinib Velexbru^®^	ONO-4059	Irreversible	CNS lymphoma, WM, CLL	2020	BTK IC_50_ = 6.8 nM
					ITK IC_50_ > 20,000 nM
					TEC IC_50_ = 48 nM
Orelabrutinib	ICP-022	Irreversible	MCL, CLL, SLL	2020	BTK IC_50_ = 1.6 nM

**Table 2:** Summary of BTKIs in clinical trials [64,75,76].

Name	Synonyms	Type of Inhibitor	Indications	Activity (IC50)
Spebrutinib	CC-292 AVL-292	Irreversible	CLL, NHL	BTK IC_50_ = 9.2 nM
				ITK IC_50_ = 1050 nM
				TEC IC_50_ = 8.4 nM
Evobrutinib	M2951 MSC-2364447C	Irreversible	RA, MS	BTK IC_50_ = 8.9 nM
Vecabrutinib	SNS-062	Reversible	CLL, SLL	BTK IC_50_ = 1.9 nM
Pirtobrutinib	LOXO-305	Reversible	CLL, MCL	BTK IC_50_ = 3.15 nM
				ITK IC_50_ > 5000 nM
				TEC IC_50_ = 1234 nM
Fenebrutinib	GDC-0853	Reversible	RA, SLE, CSU	BTK IC_50_ = 2.3 nM
				ITK IC_50_ = 1000 nM
				TEC IC_50_ = 1000 nM

CLL—chronic lymphocytic leukemia; CSU*—*chronic spontaneous urticaria; MCL—mantle cell lymphoma; MS*—*multiple sclerosis; NHL—non-Hodgkin lymphoma; RA—rheumatoid arthritis; SLE*—*systemic lupus erythematosus SLL—small lymphocytic lymphoma; IC_50_—half-maximal inhibitory concentration, the inhibitor concentration that causes a 50% decrease in enzyme activity. Various kinase activity tests are utilized to assess the selectivity of the inhibitors; hence, reported IC_50_ statistics are widely diverse; e.g., the IC_50_ values for ibrutinib against ITC range from 0.5 nM to 218 nM [5,76]. BTK—Bruton’s tyrosine kinase; ITK—interleukin-2-inducible T-cell kinase, TEC—cytoplasmic tyrosine kinase.

## Data Availability

No new data were created or analyzed in this study. Data sharing is not applicable to this article.
